# Delayed signatures of underground nuclear explosions

**DOI:** 10.1038/srep23032

**Published:** 2016-03-16

**Authors:** Charles R. Carrigan, Yunwei Sun, Steven L. Hunter, David G. Ruddle, Jeffrey L. Wagoner, Katherine B. L. Myers, Dudley F. Emer, Sigmund L. Drellack, Veraun D. Chipman

**Affiliations:** 1 Lawrence Livermore National Laboratory, Livermore, California, USA; 2 National Security Technologies, Las Vegas, Nevada, USA

## Abstract

Radionuclide signals from underground nuclear explosions (UNEs) are strongly influenced by the surrounding hydrogeologic regime. One effect of containment is delay of detonation-produced radioxenon reaching the surface as well as lengthening of its period of detectability compared to uncontained explosions. Using a field-scale tracer experiment, we evaluate important transport properties of a former UNE site. We observe the character of signals at the surface due to the migration of gases from the post-detonation chimney under realistic transport conditions. Background radon signals are found to be highly responsive to cavity pressurization suggesting that large local radon anomalies may be an indicator of a clandestine UNE. Computer simulations, using transport properties obtained from the experiment, track radioxenon isotopes in the chimney and their migration to the surface. They show that the chimney surrounded by a fractured containment regime behaves as a leaky chemical reactor regarding its effect on isotopic evolution introducing a dependence on nuclear yield not previously considered. This evolutionary model for radioxenon isotopes is validated by atmospheric observations of radioxenon from a 2013 UNE in the Democratic People’s Republic of Korea (DPRK). Our model produces results similar to isotopic observations with nuclear yields being comparable to seismic estimates.

A major step towards confirming the occurrence of an underground nuclear explosion (UNE) involves capturing short-lived noble gas radioisotopes produced by the explosion, sometimes referred to as the “smoking gun” for nuclear explosion detection^1^. Prior to entry into force of the Comprehensive Nuclear Test Ban Treaty (CTBT), concern over the ability of inspectors to capture diluted gases from deeply buried, low-yield detonations and the passage of numerous half lives before their detection during an on-site inspection (OSI)^2^ motivated a 1993 analog experiment involving a deep 1-kiloton chemical explosion. The explosion released chemical tracer gases used to calibrate an intentionally conservative model of noble gas migration, which involved only barometric pumping for vertical transport. The model was partly based on earlier work on the long-term reduction in the subsurface inventory of long-lived radioactive gases produced by UNEs at the National Nuclear Security Site due to atmospherically driven migration or barometric pumping of gases to the surface^3^, a trace-gas transport mechanism which is expected to be always present following a UNE. However, this new model focused on short-lived isotopes, such as ^133^Xe (half life: 5.24 days), which were predicted to be detectable during an OSI initiated up to several months following detonation. Other studies have also focused on barometric transport in evaluating subsurface xenon isotopic evolution^4^,^5^ or the effects of geologic and meteorologic uncertainty on arrival of gases at the surface^5^.

Actually, subsurface gas migration depends on multiple transport processes^7^,^8^ although barometric pumping is the most robust and long lived means of moving gas towards the surface. Atmospheric pressure fluctuations draw or *pull* gases from depth upward along fracture networks even when the fracture distribution is strongly anisotropic causing gases to flow more easily along a horizontal path which parallels geologic layering. On the other hand, driving or *pushing* gases to the surface by deep-seated transport processes is less effective when the permeability tensor has a dominant horizontal component. This difference between *pushing* and *pulling* gases to the surface has been used to explain why pressurization of former nuclear explosion cavities using tracer gases does not always produce rapid detections of the tracer at the surface if transport is primarily due to driving gases away from the cavity/chimney by a locally produced pressure gradient^9^. The presence of an anisotropic permeability field with large horizontal component may also explain the good agreement between the conservative model, which considered only the barometric pumping mechanism, and the 1993 analog experiment in which barometric pressure fluctuations determined the apparent arrival of tracer gases at the surface. More generally, detectable radioxenon signatures due to subsurface gas migration will depend on thermal convection resulting from the heat of detonation, barometric fluctuations and diffusion. While diffusion is negligible by itself for transporting gases over spatial scales of hundreds of meters to the surface for time scales of interest, it is transverse diffusion across fracture apertures during gas flow driven by barometric pressure fluctuations that makes “ratcheting” or barometric pumping possible, which is a well observed process^2^,^3^,^4^,^5^,^9^.

The two-decade moratorium on nuclear testing has eliminated the possibility of benefiting from well defined UNEs to evaluate time-dependent xenon isotopic signals detected locally or in the atmosphere at continental distances. Therefore, we have employed a novel tracer-gas experiment at a former UNE site along with computer simulations to develop a better understanding of radioxenon signatures captured either locally at the surface after migration along a fracture network or following a direct release of explosion cavity gases to the atmosphere. The tracer experiment was designed to evaluate the contributions to producing gas signatures at the surface by barometric pumping and thermal transport processes originating in the collapsed cavity or chimney regime, a geometry which is common to UNEs. The time-dependent loss of gases migrating from a chimney is also closely tied to the evolution of radioxenon isotopic ratios of remaining chimney gases, and we use computer simulations to track xenon isotopic evolution in the chimney. As a reality check, we compare our simulations of evolving isotopic ratios in a leaking chimney to rare isotopic measurements from atmospheric samples obtained following the 2013 DPRK UNE. This comparison also helps to illustrate the limitations on interpreting atmospheric data having observational error using computer simulations having parametric uncertainties.

Several new insights are gained from the experimental and simulation studies described here: 1) At even low pressure gradients, generally much shorter gas arrival times occurred than expected for barometric pumping alone at the tracer experiment site. 2) Our modeling shows that barometric pumping can dominate thermally driven advection only after weeks to months following a detonation. 3) Natural background radon levels were found to be anomalously high during chimney pressurization, which suggests that radon activity may potentially be a readily measured indicator of UNE-produced subsurface gas transport at a suspected test site. 4) In our simulations, we observed a novel dependence of isotopic ratios on nuclear yield due to gas transport from the chimney.

## Results

### Field Experiments

**Figure 1:**
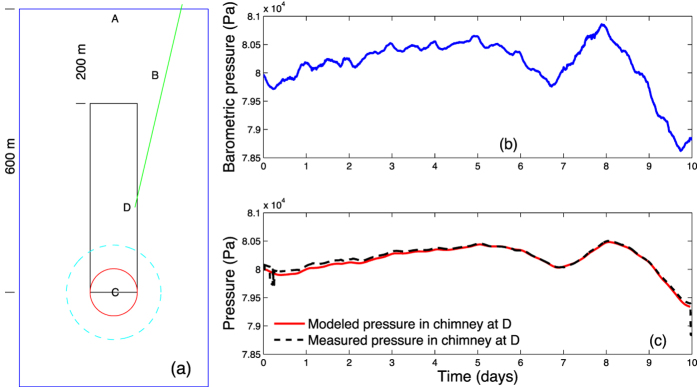
A schematic cross section of physical/simulated system and comparison of measured and modeled chimney pressure. (**a**) Model domain (width is horizontally truncated) illustrating region of detonation-produced damage and enhanced permeability. Labeled points A, B, and C in domain indicate where evolution of radioactive xenon daughters was simulated. Point D is an approximate injection point of tracer gases in chimney pressurization experiment. Red Circle represents initial cavity formation by underground explosion, and black box illustrates the chimney formed after collapse of the cavity roof^8^. The chimney consists of a rubblized rock regime resulting from the collapse. Chimney height and damage zone in simulations are scaled to explosion yield. (**b**) Barometric pressure at ground surface drives pressure changes in underlying chimney region. (**c**) Propagation of the atmospheric pressure wave through the overburden causes a distinct pressure response in the chimney. Matching the cavity fluctuations with a model, assuming measured surface pressure fluctuations as a time-dependent boundary condition, allows a best-fit estimate for the transport properties of the overlying damaged tuff (Table 1).

The tracer gas experiment used as a basis for developing both gas migration and isotopic evolution models was performed on Pahute Mesa at the Nevada National Security Site in an area having geology consisting of volcanic tuff layers with vertical cooling fractures in otherwise competent upper layers. The study site resulted from a UNE detonated in 1989 at a depth of 600 m, which produced a cavity stoping upward to form a rubble-filled chimney before terminating 200 m beneath the surface. This is the assumed geometry of the Pahute Mesa UNE model used here (Fig. 1a). Prior to continuous tracer injection of Freon 12B2 (dibromodifluoromethane) occurring during a 10-day period in 2012, background atmospheric pressure fluctuations along with the resulting temporal pressure variations in the chimney were measured, with the aid of a borehole intersecting the chimney, and used to estimate gas-transport properties at the site^9^. Figure 1b and c summarize the observations along with the best multi-parameter fit from a set of more than 1000 simulations performed to match chimney pressure fluctuations assuming a dual-porosity, fracture-matrix model representing the layers overlying the top of the chimney^8^. This is a standard approach to estimating multiple transport properties characterizing a hydrogeologic system and produces “best-fit” values of the properties that govern transport at this particular site between the chimney and surface. Table 1 gives the values of best-fit parameters that were used in subsequent simulations of transport processes at the Pahute Mesa site. Fracture aperture and spacing were found comparable to that obtained in the 1993 experiment^2^. Besides best-fit values, maximum and minimum parameter values occurring in the top 10 best fits are also included in Table 1 for comparison.

Freon 12B2 chemical tracer gas was used as an inert surrogate for radioactive xenon isotopes. For experiments intended to elucidate subsurface gas transport, chemical tracers have several significant advantages over using actual radioxenon tracers. They require that very much smaller samples be taken from the subsurface for analysis than required for analysis of radioactive xenon isotopes, typically 0.1 L versus 2000 L, a factor of 20,000 in volume. This allows both the spatial and temporal resolution of the chemical tracer to be very much higher at a given sampling site since chemical samples can be acquired as often as desired without concern for either the time required to sample or the extremely large volume of soil/fractured rock network that must be tapped to obtain a single sample (e.g., 2000 L/0.1 porosity = 20 m^3^). Additionally, acquisition of very small samples precludes the possibility of both soil gas depletion and atmospheric infiltration occurring to replace the extracted gas that is a major concern when very large samples are taken. The main advantage of chemical tracers over stable isotope tracers is that they tend to be much less expensive to deploy in this type of experiment given that relatively large quantities of stable isotope must be injected into the chimney to overcome the significant levels of the stable isotopes already present in soil gas and the atmosphere. Of course, physical property differences between the surrogate and radioxenon that might affect transport, such as binary diffusivity and solubility in groundwater, must be taken into account usually through post-experiment modeling as was done for the 1993 analog experiment^2^ and in this study.

The continuous injection of tracer mixed with air (∼47 m^3^min^−1^ at 1 bar) into the chimney (3 × 10^5^ m^3^) achieved a measured pressurization of about 40 mbar simulating the post-detonation, thermal convection phase of a UNE, a process not considered in previous studies. This chimney-to-surface pressure difference is approximately equivalent to that of a large tropical storm providing only 10 ∼20% of the effective drive associated with thermally driven convection according to our transport simulations. At the site, signals produced by chimney pressurization and barometric pumping were monitored using a new, automated, high-temporal resolution soil-gas sampling system, the Subsurface Gas Smart Sampler (SGSS)^7^, representing a major improvement over often sporadic, low-resolution manual sampling used earlier^2^.

**Figure 2:**
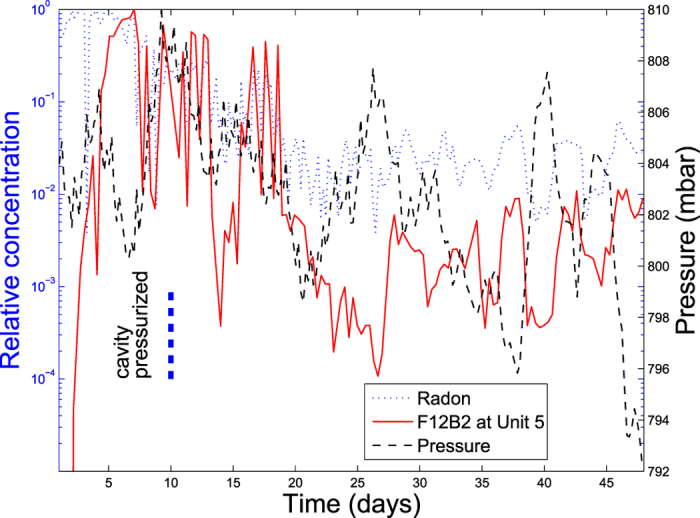
Concentrations of Freon 12B2 at SGZ (station #5) normalized by the maximum concentration measured in the time history. Soil gas samples of 0.1 to 0.5 L were captured beneath a tarpaulin while measuring barometric pressure and background radon concentration. Four other sampling sites produced similar results, but only SGZ data are shown here to reduce the complexity of the plot. The highest concentration occurs during the first 10 days when the chimney is weakly pressurized (∼40 mbar) by pumping air into the borehole with tracer. Sampling was performed either by extracting gases directly from the subsurface at depths of about 3 m or by taking soil gas directly from beneath tarpaulins (∼3 m × 20 m) spread over a crack or similar feature. First detected arrival occurs at SGZ about 2 days after the start of pressurization. Following the 10-day period of pressurization, all sites continue to produce high levels of tracer. The tracking of radon, normalized by highest activity measured, with tracer concentration during chimney pressurization is apparent as measured radon levels are at least 10 ∼15 fold higher during the pressurization period.

Five sampling stations were monitored and all produced similar results except for variations in tracer arrival times and concentrations. Figure 2 illustrates breakthrough for only the surface-ground-zero (SGZ) sampling station (#5). The earliest initial breakthrough (solid red line) followed by very high levels of tracer (concentration >10% of chimney values) was observed in less than 2 days at SGZ, again for a pressure gradient of only 10 ∼20% of that expected for thermally driven convection. Breakthrough occurred at all stations during the pressurization period. By comparison, breakthrough involving only barometric pumping was predicted to require months according to our models of the Pahute Mesa site using the transport parameters of Table 1. The fracture regime at this site includes deep trending vertical cooling fractures in the volcanics. We speculate this would favor thermally driven transport over barometric transport if the earlier argument^9^ is valid regarding the need for much higher horizontal than vertical permeability for barometric transport to control tracer arrivals.

During the 10-day pressurization, barometric effects still modulated concentrations almost 100 fold and caused tracer at another station to be detected during falling pressure and then vanish when atmospheric pressure rose only to reappear when pressure decreased. This suggests that even during pressure-dominated transport, barometric effects can still be important. During the pressurization period, background radon levels were observed to exceed post-pressurization levels by factors of 10 ∼15 (Fig. 2) suggesting that local background radon anomalies could be an easily measured indicator of a UNE-pressurized subsurface flow regime that cannot otherwise be immediately detected. It was found that soil-gas tracer concentrations generally exhibited a good correlation with measured background soil gas radon levels and were anti-correlated with barometric pressure as illustrated in Fig. 2. It should be noted that both barometric pressure and radon monitoring were done at much higher temporal resolution than chemical tracer sampling (1 sample/8 hours) and only broad comparisons of the correlations with the tracer concentration history are possible. However, comparison between the barometric and radon histories is possible. For the case of the sampling site at SGZ, the peaks and valleys (Fig. 2) of the radon history correlate virtually perfectly (*R*^2^ = 0.99984) with barometric peaks and valleys if the radon history is shifted to lag the barometric history by approximately 2.3 hours at this particular site. This lag is apparently the finite response time required by the tarpaulin-covered fracture regime for producing radon captured in the samples from beneath the tarp. Since the system does not respond instantaneously to pressure fluctuations, taking into account this type of delay during sampling within a period of falling barometric pressure may enhance concentrations of gases that are captured. This suggests background radon levels and fluctuations might be studied under more general conditions in conjunction with barometric pressure fluctuations as possible indicators for triggering sample collection when obtaining maximum tracer or noble gas concentrations is required. Unlike barometric pressure fluctuations that are a trigger for sampling only when barometric pumping is dominant, radon fluctuations would be expected to be correlated with the vertical flux of tracer driven by all subsurface transport processes.

## Mathematical Models

Our field experiment deals only with the transport of a surrogate chemical tracer rather than an evolving suite of parent/daughter radionuclides having different migration characteristics in the subsurface. Using the hydrogeologic characterization of the Pahute Mesa site obtained from the field experiment in a two-phase convection model with barometric pumping, we developed computer simulations using the NUFT^10^,^11^ simulator that take into account both the production of radioxenon daughter products from their radionuclide parents^17^ within the post-detonation chimney as well as the effect of differential migration associated with high-mobility noble gas daughters and the low-mobility chemically reactive iodine parent. Evolution of the full isotopic makeup of a noble gas signature was evaluated at several locations within the containment regime using a radionuclide decay-chain/network computational model for simultaneously tracking 22-isotope parent/daughter radionuclides in the presence of subsurface transport processes^12^. Simulations included effects of differential subsurface migration of parent/daughter isotopes and partitioning of gases between air and water at the pore scale. It was previously shown that neglecting differential migration of parent and daughter isotopes in the subsurface may result in mis-interpretation of detonation-produced xenon ratios as civilian nuclear activities (e.g., medical isotope production) during the first 10 days following a UNE^4^,^12^.

**Figure 3:**
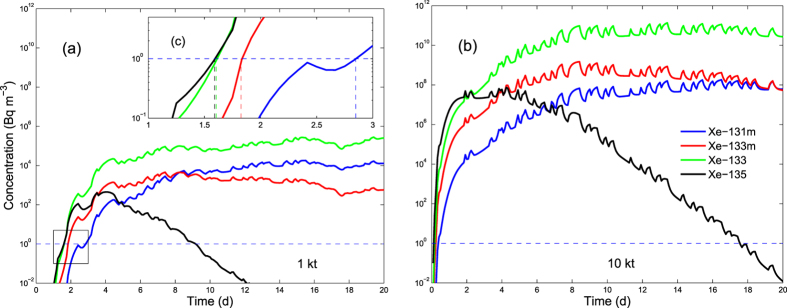
Simulated activity concentrations of xenon isotopes for 1 (**a**) and 10 kilotons (**b**), with four xenon isotopes arriving above the detection limit at different times. Subplot (**c**), zoomed plot showing details of isotopic arrivals. In addition to fracture properties as given in Table 1, we simulated heat-pipe transport resulting from the heat of detonation and phase partitioning between pore gas and liquid. The arrival of xenon isotopes occurs much earlier following detonation than for barometric pumping alone.

Simulations of arrival times at SGZ (Fig. 1a, location A) for different nuclear yields were calculated for several radioxenon isotopes. Typical arrivals at the 1 Bq m^−3^ level are less than 2 days for a 1 kiloton event, comparable to the result obtained from the experiment, and 0.5 days for 10 kilotons (Fig. 3). It is also seen that significant modulation of the isotopic concentration by barometric fluctuations occurs at progressively later times for increasing yields. In our simulations, barometric pumping dominates transport by heat-pipe and other mechanisms only after several weeks to months.

Besides providing a basis for interpreting noble gas sampling at the surface or while performing drilling during an OSI, we argue that simulating the spatial dependence of xenon isotopic ratios in the subsurface is potentially valuable for interpreting observations of remotely detected noble gases by the International Monitoring System (IMS). Noble gases collecting either in a cavity/chimney system or in nearby access tunnels evolve distinctive isotopic signatures^13^. Bulk releases of these gases into the atmosphere by opening tunnels or drilling back into post-detonation chimneys, a common practice, are detectable under certain conditions.

**Figure 4:**
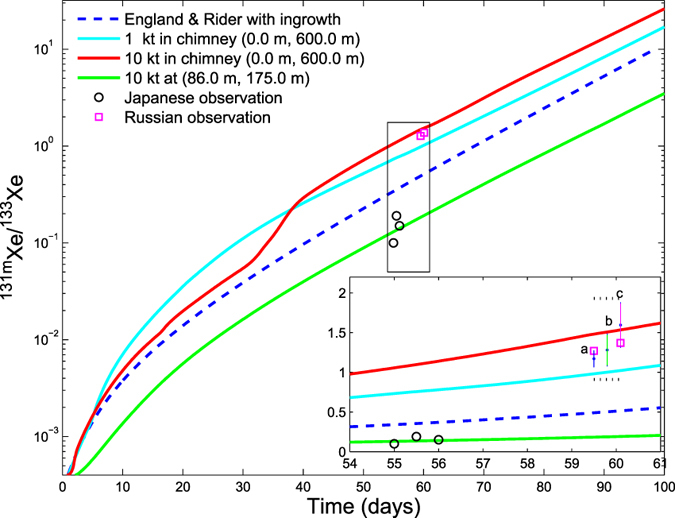
Comparison of simulated and measured activity-concentration ratios of ^131m^Xe/133 Xe as a function of time since detonation. The dashed line is the prediction consistent with England and Rider assuming all parent/daughter isotopes remain in a well-mixed chimney. Red and blue lines correspond to ^131m^Xe/^133^ Xe evolutionary paths appropriate for 10-kiloton and 1-kiloton detonations in a volcanic zone, respectively. The geometry of the damage models (e.g., enhanced permeability out to two-cavity radii) is based on field observations from previous underground nuclear explosions and assumes transport-property values consistent with the present study (Table 1). The inset magnifies the region on the Pahute Mesa evolutionary curves containing the Russian and Japanese observations of isotopic ratios. For comparison to the Pahute Mesa results in volcanics, we also include a parameter-sensitivity study involving variations in permeability and nuclear yield in a hard rock zone that is more representative of the DPRK test site assuming a 430 m depth of detonation^15^. The ranges are 1 to 10 × 10^−12^ m^2^ for permeability, which spans estimated damage permeabilities for explosions in granite^16^, and (a) 1 to 3, (b) 5 to 8, and (c) 9 to 10 kilotons for yield. 1*σ* measurement errors estimated by Ringbom *et al*.^13^ and given by dotted horizontal lines are found to be comparable to the parametric uncertainties of the simulated DPRK event. The green line is the evolutionary path for gases monitored at tunnel location B (Fig. 1a). Square symbols indicate ratios observed at Russian (two clustered higher-lying squares) and Japanese (3 clustered lower-lying circles) IMS radionuclide stations following the February 2013 event^13^. Cross-over of the evolutionary paths for the 1- and 10-kiloton cases is a result of the sublinear scaling of thermally driven convection with yield-dependent heating while the production of radioxenon isotopes varies linearly with yield.

Our simulations of differences in migration between parent and daughters on the longer term (>10 days) due to convection-driven leakage from the chimney and differential migration result in evolutionary histories for the radioxenon isotopes shown in Fig. 4 represented by the ^131m^Xe/^133^Xe ratio for 1 and 10 kiloton UNEs. The curves correspond to locations shown in Fig. 1a (cavity/chimney-C; B-86 m distant from SGZ, depth of 175 m) and we find that in tunnels or voids near the surface, the ratio evolves quite differently from the ratio in the chimney itself. Primarily for the purpose of validating our simulations, we also include multiple IMS measurements of this ratio determined from atmospheric samples detected first in Japan and several days later in Russia in April 2013 and associated with the February 2013 declared DPRK UNE at the Punggye-ri Nuclear Test Site, which have been analyzed in detail by Ringbom *et al*.^13^.

The two higher Russian measurements of this ratio lie close to the evolutionary track associated with 10-kiloton-yield chimney gas in our model, consistent with the volcanic material of Pahute Mesa, while the three lower-lying Japanese measurements are intersected by an evolutionary line calculated for a void or tunnel at location B in Fig. 1a. Our simulation of isotopic ratio evolution supports the qualitative interpretation of Ringbom *et al*. that the Russian detections involve gas releases from deeper within the UNE site. In fact, our modeling suggests more specifically that the Russian detections are a result of gases released directly from the explosion cavity/chimney given the correspondence between observed and simulated isotopic ratios at the time of detection. Additionally, the Russian observations are bounded by the 1- and 10-kiloton predictions of our model in agreement with seismic yield estimates^13^,^14^ of the February 2013 event.

However, significant uncertainties surround the atmospheric observations^13^, containment parameters used in the evolutionary model and the seismic estimates. In the inset of Fig. 4, we show the effect of these uncertainties and estimated errors. First, the estimated (1*σ*) error for the observed isotopic ratios^13^ is given by the two horizontal dotted lines. The 1*σ* error clearly spans the 1 ∼10 kiloton estimates of the model appropriate to Pahute Mesa. For comparison, we also performed a number of random parameter simulations involving a range of nuclear yields and post-detonation permeabilities (1 ∼10 × 10^−12^ m^2^) to estimate the effect of uncertainty on simulated isotopic ratio for containment parameters more appropriate to the DPRK UNE. These simulations assume an estimate of detonation depth (430 m)^15^ and bound an estimate of the range of post-detonation permeabilities of the the granitic DPRK test site (1 ∼3 × 10^−12^ m^2^)^16^. Three distinct ranges of nuclear yield are simulated between 1 and 10 kilotons represented by the three vertical lines (a–c) which span a range of variation in isotopic ratio associated with each range of nuclear yield. Line b, corresponding to the 5 to 8 kiloton range of simulations, is the only yield range capturing both Russian observations. However, we find that the 1*σ* error of the Russian measurements again spans the full modeled range of parameter uncertainty for the lines (a, b, and c) corresponding to a simulated yield of 1 ∼10 kilotons. Assuming continuation of the trend of increasing values of isotopic ratio with increasing yield as shown by lines a to c, simulations with yields larger than 10 kilotons (line c) will begin to fall outside the upper 1*σ* error bar of the Russian observations. We interpret this trend to mean that UNE scenarios involving higher yields than 10 kilotons are likely to fit the observed Russian observations less well, while scenarios less than 10 kilotons yield are more consistent with these observations.

## Discussion

An implication for an OSI from our high-resolution sampling and modeling experiments on Pahute Mesa is the potential dominance over barometric pumping at early times (weeks to months) of gas transport by chimney-driven processes resulting in more rapid and larger amplitude noble gas arrivals (hours to a few days) at the surface for even well contained events suggesting the value of initiating an inspection as soon as possible to obtain the largest subsurface signals. While barometric pumping becomes gradually more important in the weeks following detonation as multiphase transport wanes, we found it can still strongly modulate the concentration of captured gases at early times implying that sampling during falling barometric pressure should still be considered for maximizing noble gas concentrations in samples.

Monitoring radon background during the sampling process has previously been considered as a means of determining if atmospheric air (low radon) is leaking into gas samples extracted from the subsurface as indicated by rapidly decreasing radon levels during monitoring of gas sampling operations. Mixing of atmospheric gas with subsurface gas is a source of dilution and possible contamination if isotopic radioxenon is already present in the atmosphere from nearby nuclear reactors or medical isotope facilities. However, our results suggest a broader application for background radon monitoring given that very large radon anomalies were observed over the site during weak pressurization of the chimney. Large radon anomalies may be strong indicators for thermally driven processes associated with a UNE. We may also use the lag time between radon measurements and barometric fluctuations to determine the best times to obtain a soil gas sample with maximum noble gas concentration, although this application requires a better understanding of the connection between subsurface radon transport and the migration of chimney gases.

Extensive fracturing along with physicochemical differences in the migratory ability of different components of the decay chain violates the assumptions of the idealized radioisotopic model of England and Rider^17^ for the evolution of radioxenon isotopes. It therefore cannot apply to general containment scenarios where chimney leakage occurs. Because of differential migration and spatial differences in transport processes, we also find that noble gas amplitudes and chemical makeup (i.e., isotopic ratios) vary over the surface and in subsurface voids such as tunnels. We present the first numerical simulations of post-detonation, xenon isotopic evolution in a degassing chimney regime subject to both multiphase convection and barometric pumping using the Pahute Mesa model, which is based on our experimental observations. Such simulations of subsurface isotopic evolution are highly relevant to often proposed drilling by CTBT inspectors into a UNE cavity/chimney regime for gas sampling purposes during an OSI.

We also modify the isotopic evolution model to simulate a granitic geologic regime involving a range of different nuclear yields to compare it with atmospheric measurements of radioxenon following the February 2013 DPRK underground nuclear explosion. The results are consistent with a gas-release scenario explained by opening an outer access tunnel, which produces the first release of radioxenon a few days before completion of a drill back hole into the explosion cavity, which corresponds to the second release, consistent with the more qualitative model of Ringbom *et al*.^13^. The dependence of the simulated isotopic ratio on nuclear yield is related to the ability of yield-dependent convection to flush isotopic gases from the explosion cavity at early times. While the range of yields (5 to 8 kilotons) required by our model to qualitatively bound observed isotopic ratios is comparable to seismic estimates of the February 2013 event, additional sensitivity studies are needed to assess the applicability of this approach to the estimation of the nuclear yield of future underground nuclear explosions. Finally, our results suggest the value of reducing atmospheric measurement errors of isotopic ratios if additional insight into a detonation responsible for the observed noble gas isotopic ratios is to be achieved. Insights gained from the interaction of transport processes affecting delayed signals are also potentially applicable to monitoring other engineered subsurface regimes, which are either thermally or mechanically pressurized, such as *in situ* coal gasification, deep sequestration of CO_2_ and nuclear waste disposal.

## Methods

### Sampling locations

**Figure 5:**
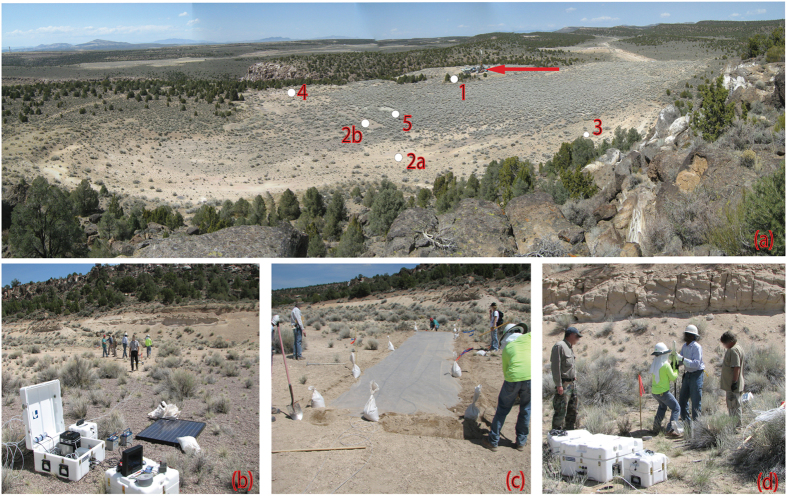
(**a**) Experiment site. Site 1 is a small tarp serviced by one sampler. Site 2 is one sampler servicing two tarps (**a,b**). Site 3 (see also Fig. 5d) is one sampler servicing several tubes inserted in the ground close together by nearby bluff. Site 4 is one sampler servicing tube inserted in fracture. Site 5 (SGZ) is one sampler servicing tarp laid over cement-filled emplacement shaft. The red arrow indicates the location of the drill-back hole. (**b**) Automated sampler as deployed at Site 2. (**c**) Tarpaulin installed at Site 2a. (**d**) Emplacing sample tubes at Site 3. (Fig. 5 photos: Sigmund Drellack).

Shown in Fig. 5a, all five sampling sites are located within 175 m of surface ground zero (SGZ). Soil gas at SGZ site is extracted using 0.64 cm (0.25 in) perforated tubing placed beneath a tarpaulin covering concrete-filled emplacement borehole. No cracks or fractures were observed in the concrete fill. Remaining sites utilize tarpaulins or sampling tubes inserted to shallow depth (∼2–3 m) where possible cooling fractures are covered by thin soil layer (<0.2 m). Figure 5b, automated samplers are solar powered and operate in a continuous mode extracting soil gas at approximately 0.5 L min^−1^ from beneath tarps or from shallow sampling tubes. Typical soil gas sampling frequency is 1 per 8 hours and sample volume is 0.1 to 0.3 L. In the continuous mode, radon background is measured by the sampler 4 times per hour. In Fig. 5c where tarpaulins are deployed, edges of a tarp are covered with mounded soil to reduce infiltration. Deployment of shallow sampling tubes (Fig. 5d) includes sealing with bentonite around tube in soil to prevent direct infiltration of air to the subsurface sampling point. Following insertion of the tube, the surface around the sampling site is covered with a tarp to further minimize atmospheric infiltration during the monitoring process.

Tracer injection parameters are provided in Table 2. The injection system was designed to permit co-injection of chemical tracer with atmospheric air. The continuous 10-day injection produced a pressure gradient less than expected for thermally driven multiphase convection according to our models.

### Gas detection

The sampling site at SGZ showed earliest detectable tracer arrival approximately 2 days following initiation of chimney pressurization. Maximum observed tracer concentration at SGZ was 10% of chimney level. Detectable tracer levels were observed at all sites within the 10-day pressurization period. All sites experienced similar anti-correlated responses between barometric pressure fluctuations and tracer concentrations and correlated responses between background radon concentrations and tracer concentrations.

### Physics

Non-isothermal multiphase reactive transport is mathematically expressed by mass and energy balance equations for water, air, and 22 radionuclides in liquid, gas, and non-deformable solid phases. Water and air are the major components in the liquid and gas phases while 22 radionuclides are minor components with relatively-low mass fractions in two fluid phases. Four physical processes (thermally-driven advection and dispersion, barometric-pumping induced advection, matrix diffusion, and chain reactions) determine the xenon signatures observable at the surface. The physics of thermally-driven advection and heat-pipe phenomena observed and interpreted by Sun *et al*.^18^ are the major contributors to the early detection of xenon signals following a UNE. Barometric pumping or ratcheting partially contributes to gas arrivals at early time following the detonation. As the explosion-residual heat declines, barometric pumping increases its role in transporting relatively long-life isotopes.

### Radiochemistry

Radioactive decay and ingrowth are based on England and Rider^17^. As fission products, xenon isotopes are formed directly (as independent yields expressed as initial conditions) from fissions and through successive chain reactions^12^,^19^. The radioactive decay networks are given by Fig. 1 of Sun *et al*.^12^. While parent species may be assumed to stay in the solid and liquid phases remaining well contained in the chimney^7^, xenon isotopes are produced and transported in the gas phase from a detonation point to the ground surface and atmosphere. Then, xenon isotopes or their daughter products can be sampled at the ground surface or in the atmosphere away from the detonation location for detection purposes.

### Numerical simulations

The coupled physical and radiochemical processes are modeled using the USNT module of the NUFT simulator^10^,^11^ developed at Lawrence Livermore National Laboratory. The models are developed in a two-dimensional, radial-symmetric domain extending vertically from the ground surface to the water table and radially from the vertical axis passing through the detonation point to the outer boundary at a significant distance where the assumption of the ambient thermal and concentration condition is justified. We assume a dependence of cavity radius on yield, in common with another study^6^, where radius is calculated as a cube-root function of yield^20^. However, instead of using the spherical cavity geometry in our simulations, we calculate the dimensions of a cylindrical chimney commonly resulting from the collapse of the cavity as the initial source domain.

At the ground surface boundary, gas-phase conditions are specified by the air composition and the liquid-phase saturation is fixed to be zero. At the water table boundary, liquid-phase conditions are specified based on groundwater composition and full-liquid saturation. The measured air pressure with daily and hourly variations is set at the ground surface as a time-varying boundary condition. Hydrostatic initial conditions between ground surface and water table are established by running the pre-simulation model using the specified boundary conditions, physical properties, and stratigraphy.

The fractured rock system is conceptualized as a fracture-matrix dual-permeability medium and the interaction between fractures and matrix is characterized based on statistical measures of fracture-aperture size, orientation, and frequency.

### Uncertainty quantification

We employ the PSUADE^21^, non-intrusive or sampling-based approach, for calibrating system parameters and conducting sensitivity analyses. Latin hypercube sampling^22^ is used to generate sample points in the parametric space defined by the range of uncertainty for each parameter. NUFT models are then developed and executed at these sample points for evaluating objective function and constraints for system calibration. Using measured pressure data and Sobol’ sensitivity analysis^23^,^24^, fracture aperture size, frequency, and permeability are found to be sensitive parameters.

## Additional Information

**How to cite this article**: Carrigan, C. R. *et al*. Delayed signatures of underground nuclear explosions. *Sci. Rep*. **6**, 23032; doi: 10.1038/srep23032 (2016).

## Figures and Tables

**Table 1:** System parameter ranges and best fit.

Parameter	Unit	Minimum	Maximum	Mean	Optimized
Fracture aperture	[m]	1.10 × 10^−4^	8.95 × 10^−3^	(9.98 × 10^−4^)	1.48 × 10^−3^
Fracture frequency	[–]	1.284	9.460	3.943	3.996
Fracture permeability	[m^2^]	9.26 × 10^−11^	7.52 × 10^−9^	(1.37 × 10^−9^)	6.96 × 10^−10^
Bulk permeability	[m^2^]	3.06 × 10^−12^	6.47 × 10^−12^	(4.46 × 10^−12^)	4.13 × 10^−12^

Numbers in parentheses indicate the mean values on log_10_ scale.

Fracture and matrix porosities are assumed to be 0.99 and 0.1.

**Table 2:** Tracer injection parameters.

Parameter	Value
Injection period	10 days
Injection depth	∼450 m
Average air injection rate	47 m^3^ min^−1^ (2100 moles min^−1^)
Chemical tracer	Freon 12B2 (molecular weight, 210)
Co-injection rate	∼0.0303 kg min^−1^ (0.144 moles min^−1^)
Average injected tracer concentration	6.85 × 10^−5^ (68.5 ppm)
Chimney pressurization during injection	40 mbar
